# Immune cell death-related lncRNA signature as a predictive factor of clinical outcomes and immune checkpoints in gastric cancer

**DOI:** 10.3389/fphar.2023.1162995

**Published:** 2023-04-04

**Authors:** Zeyu Zhang, Duntao Su, Abhimanyu Thakur, Kui Zhang, Fada Xia, Yuanliang Yan

**Affiliations:** ^1^ Department of General Surgery, Xiangya Hospital, Central South University, Changsha, Hunan, China; ^2^ Pritzker School of Molecular Engineering, Ben May Department for Cancer Research, University of Chicago, Chicago, IL, United States; ^3^ State Key Laboratory of Silkworm Genome Biology, Medical Research Institute, Southwest University, Chongqing, China; ^4^ Department of Pharmacy, Xiangya Hospital, Central South University, Changsha, Hunan, China; ^5^ National Clinical Research Center for Geriatric Disorders, Xiangya Hospital, Central South University, Changsha, Hunan, China

**Keywords:** gastric cancer, immune cell death, lncRNAs, prognostic signature, tumor immune microenvironment

## Abstract

**Background:** Immune cell death (ICD) is a type of tumor cell death that has recently been shown to activate and regulate tumor immunity. However, the role of ICD-related long non-coding RNAs (lncRNAs) in gastric cancer remains to be clarified.

**Methods:** We obtained 375 tumor samples from the Cancer Genome Atlas (TCGA) database and randomly assigned them to training and verification groups. LASSO and Cox regression analysis were utilized to identify ICD-related lncRNAs and establish a risk model. The changes in the immune microenvironment of the two groups were compared by examining the tumor-infiltrating immune cells.

**Results:** We established a tumor signature based on nine ICD-related lncRNAs. In light of the receiver operating characteristic and Kaplan–Meier curves, the prognostic values of this risk model were verified. Multivariate regression analysis showed that the risk score was an independent risk factor for the prognosis of patients in both the training cohort (HR 2.52; 95% CI: 1.65–3.87) and validation cohort (HR 2.70; 95% CI: 1.54–4.8). A nomogram was developed to predict the 1-, 3-, and 5-year survival of patients with gastric cancer, and the signature was linked to high levels of immunological checkpoint expression (B7-H3, VSIR).

**Conclusions:** An ICD-related lncRNA signature could predict the immune response and prognosis of patients with gastric cancer. This prognostic signature could be employed to independently monitor the efficacy of immunotherapy for gastric cancer patients.

## Background

Gastric cancer is the fifth most prevalent malignant cancer in the world, with the fourth highest mortality rate (Gao et al., 2022). Gastric cancers are usually at advanced stages when diagnosed, and the prognosis is poor (Sung et al., 2021). The main treatment methods for gastric cancer are surgery, radiotherapy, and chemotherapy (Fujiyoshi et al., 2021). In spite of the breakthroughs in immunotherapy in the past years (Wang et al., 2021a), the prognosis for gastric cancer patients remains poor. Thus, new biomarkers and preclinical models should be developed to identify more targets for the treatment and prognosis of gastric cancer.

Immune cell death (ICD) is a form of tumor cell death that has recently been revealed to activate and regulate tumor immunity. ICD promotes the recovery of a dysregulated antitumor immune system in the tumor microenvironment (TME). When ICD occurs, damage-associated molecular patterns (DAMPs) are released, which interact with dendritic cells (DCs) and promote their maturation. This improves the processing of engulfed cells and antigen presentation by DCs. DCs can stimulate certain T lymphocytes to exert cytotoxic effects on tumor cells *via* the mediation of antigen-presenting cells. ICD can eventually result in durable anticancer immunity in the host (Zhou et al., 2019). ICD-based anticancer drugs, such as belantamab, mafodotin (Tzogani et al., 2021), and lurbinectedin (Markham, 2020), are available for the treatment of melanoma and small cell lung cancer, respectively; however, ICD-related therapies for gastric cancer are rare. Further research is required to identify relevant biomarkers and conduct preclinical investigations.

Long non-coding RNAs (lncRNAs) are a type of RNA approximately 200 nucleotides in length. Due to their great specificity, they can be collected non-invasively, and their capacity to adapt to pathological and physiological changes in the human body makes them a research hotspot. LncRNAs are potential diagnostic and prognostic biomarkers for numerous diseases, and they can also influence the biological activity of cancer cells, including proliferation, migration, and invasion (Zhou et al., 2022). Recent research has demonstrated that lncRNAs can be utilized as reliable molecular markers for gastric cancer identification and progression prediction. LncRNAs may also aid in identifying new treatment and prognostic targets for gastric cancer (Fattahi et al., 2020).

We developed an ICD-associated lncRNA risk model that has good predictive value for the immune responsiveness and prognosis of patients with gastric cancer. This risk model could be utilized for the clinical management of patients with gastric cancer.

## Materials and methods

### Datasets and clinical information

For the purposes of this study, clinicopathological characteristics of gastric cancer and RNA sequence information were extracted from the Cancer Genome Atlas (TCGA, https://portal.gdc.cancer.gov/). Three patients were excluded due to incomplete clinical information. A total of 372 gastric tumor tissues and 32 normal tissues were obtained.

### Identification of ICD-related lncRNAs

Based on previous research, 138 ICD-related genes were identified (Zhang and Chen, 2022). Pearson correlation analysis was performed to determine the association between lncRNAs and ICD-related genes. The criteria for Pearson correlation were a coefficient >0.4 and *p*<0.001.

### Development and validation of a prognostic ICD-related lncRNA signature

Tumor samples were randomly divided into a training group and a verification group at a ratio of 1:2. The training cohort was used to build a risk model for ICD-related lncRNA, and the validation cohort was used to verify the risk model. First, univariate and multivariate Cox regression analyses were performed to identify prognosis-related lncRNAs. Then, ICD-related lncRNAs were screened using LASSO (least absolute shrinkage and selection operator)-based Cox regression. We calculated the risk score of the patients according to the following formula: risk score = expression of lncRNA1 b1lncRNA1 + expression of lncRNA2 b2lncRNA2 + expression of lncRNA bnlncRNAn. Based on the median risk score, the samples were assigned to a high-risk group or a low-risk group, and the effectiveness of the risk model was verified by receiver operating characteristic (ROC) and Kaplan–Meier (K-M) curves. Lastly, a nomogram was constructed to predict gastric cancer prognosis based on sample data.

### The mRNA–lncRNA co-expression network

A co-expression network was constructed to show the relationship between ICD-related genes and ICD-related lncRNAs, and a Sankey diagram was created to depict the relationship among lncRNAs, mRNAs, and risk types.

### GSEA and functional enrichment analysis

The ‘limma’ package was used to identify differentially expressed genes between normal and tumor tissues with cutoff criteria of <0.05 for the false discovery rate and >1 for |log2foldchange|. The gene expression data were interpreted by gene set enrichment analysis (GSEA) (http://www.broadinstitute.org/gsea) (Subramanian et al., 2005), and CIBERSORT (Barbie et al., 2009) was used to illustrate the tumor-infiltrating immune cells in gastric cancer.

### Statistical analysis

Data analysis was performed using R, version 3.30. The *t*-test and Wilcoxon test were conducted for group comparisons, and one-way analysis of variance (ANOVA) was performed to compare the two samples. The model’s prognostic value was evaluated by univariate and multivariate regression analyses, with *p*< 0.05 being considered statistically significant.

## Results

### The identification of ICD-related prognostic lncRNAs in normal and gastric cancer tissues

**FIGURE 1:**
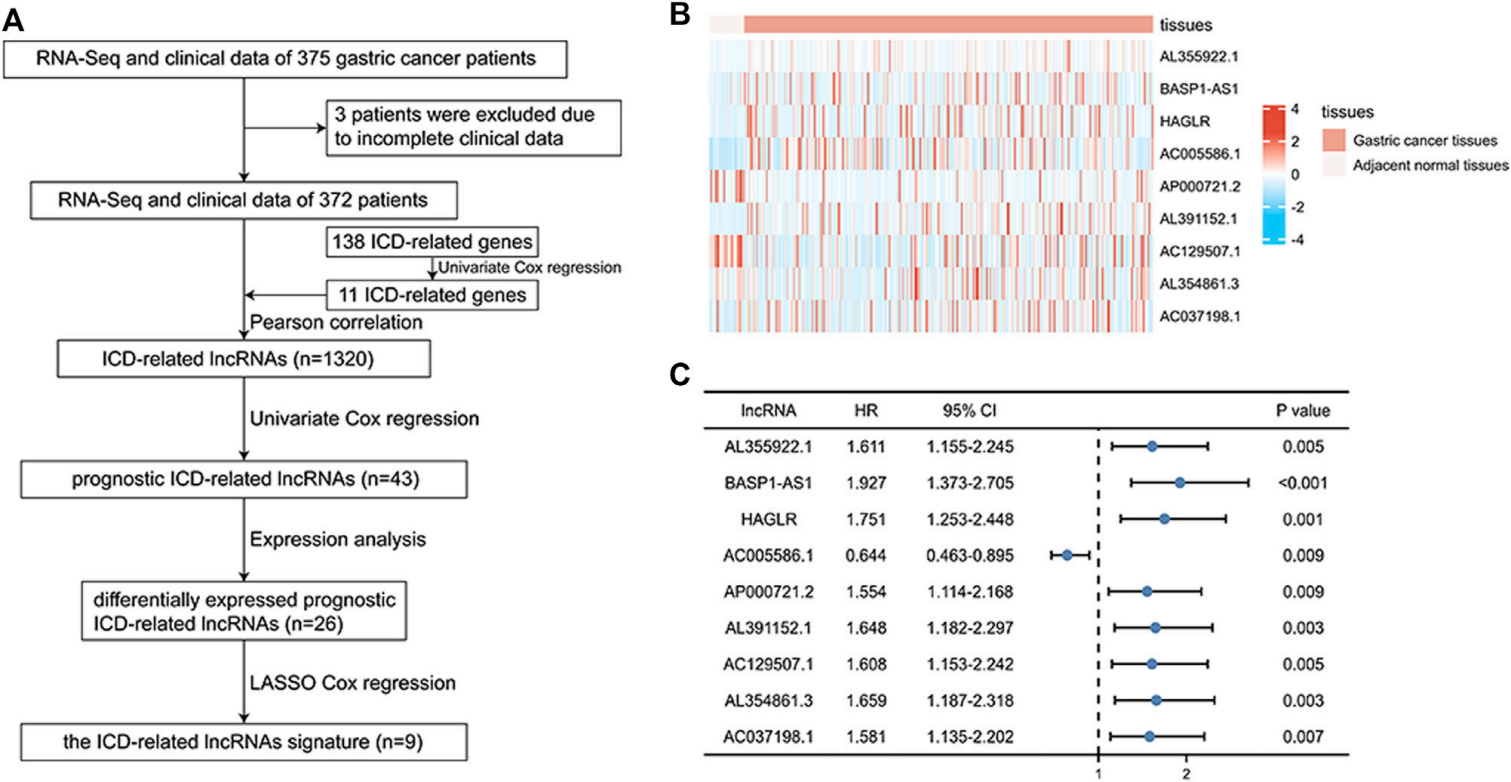
Identification of ICD-related prognostic lncRNAs between normal and gastric cancer tissue. **(A)** Flowchart for construction of the ICD-related lncRNA signature. **(B)** Heatmap of nine prognostic ICD-related lncRNAs in gastric cancer tissues and adjacent normal tissues. **(C)** Univariate Cox regression of nine prognostic ICD-related lncRNAs. *p < 0.05; **p < 0.01; ***p < 0.001.

**FIGURE 2:**
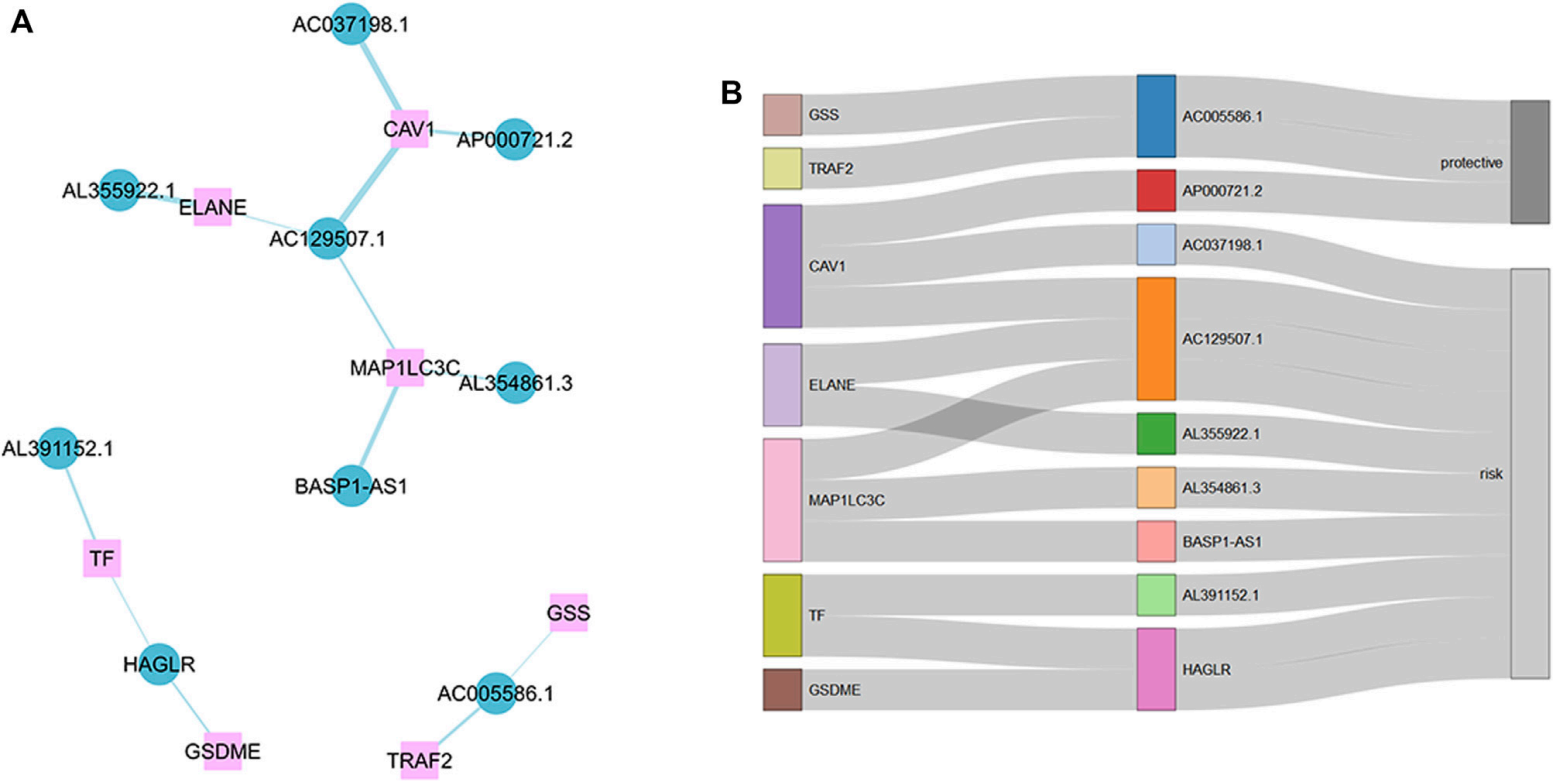
Messenger RNA (mRNA)–lncRNA co-expression network. **(A)** mRNA–lncRNA co-expression network of the ICD-related genes and selected ICD-related lncRNAs. **(B)** Sankey diagram showing connection degree between ICD-related lncRNAs and ICD-related genes.

First, we collected data on gastric cancer from the TCGA database and obtained RNA sequences and clinicopathological information from 375 tumor tissues and 32 normal tissues. This sample set was randomly divided into a training cohort and a verification cohort in a ratio of 1:2. The characteristics of the samples in the two cohorts are shown in Table 1. It can be seen that there are almost no significant differences between the two cohorts. There were 138 ICD-related genes acquired from previous studies (Zhang and Chen, 2022; Supplementary Table S1). Eleven prognostic ICD-related genes were identified by univariate Cox regression analysis, and we obtained 1,320 ICD-related lncRNAs using Pearson correlation analysis. After repeated univariate Cox regression analyses, it was found that 43 ICD-related lncRNAs were associated with gastric cancer prognosis. The expression analysis results showed that 26 prognostic ICD-related lncRNAs were differentially expressed in the two datasets. Lastly, nine ICD-related lncRNAs were used to construct the signature by LASSO Cox regression. The operational flow chart is shown in Figure 1A, and the expression levels of the nine ICD-related lncRNAs in gastric cancer and adjacent normal tissues are shown in Figure 1B. The univariate Cox regression analysis results are shown in Figure 1C. In Figure 2A, the mRNA–lncRNA co-expression network illustrates the relationship between mRNA and lncRNA. For example, AC129507.1 is related to three mRNAs: CAV1, ELANE, and MAP1LC3C. The Sankey diagram in Figure 2B illustrates the relationship among ICD-related mRNA, lncRNA, and risk types. We found that AC005586.1 and AP00072102 exerted inhibitory effects against gastric tumors.

**TABLE 1:** Characteristics of gastric cancer patients in the training cohort and validation cohort.

Characteristic	Training	Validation	*p*	Statistic
n	248	124		
Status, n (%)			0.792	0.07
Alive	153 (61.7%)	74 (59.7%)		
Dead	95 (38.3%)	50 (40.3%)		
Gender, n (%)			0.379	0.77
Female	93 (37.5%)	40 (32.3%)		
Male	155 (62.5%)	84 (67.7%)		
Race, n (%)			0.896	
Asian	50 (23.4%)	23 (21.1%)		
Black or African–American	7 (3.3%)	4 (3.7%)		
Native Hawaiian or other Pacific islander	1 (0.5%)	0 (0%)		
White	156 (72.9%)	82 (75.2%)		
Neoplasm histologic grade, n (%)			0.342	
G1	5 (2.1%)	5 (4.1%)		
G2	86 (35.5%)	48 (39.7%)		
G3	151 (62.4%)	68 (56.2%)		
Stage event pathologic stage, n (%)			0.766	1.14
Stage I	32 (13.7%)	18 (15.7%)		
Stage II	73 (31.2%)	38 (33%)		
Stage III	105 (44.9%)	45 (39.1%)		
Stage IV	24 (10.3%)	14 (12.2%)		
Stage pathologic T, n (%)			0.695	1.45
T1	13 (5.3%)	5 (4.1%)		
T2	48 (19.8%)	30 (24.8%)		
T3	113 (46.5%)	55 (45.5%)		
T4	69 (28.4%)	31 (25.6%)		
Stage pathologic N, n (%)			0.365	3.18
N0	71 (30.2%)	37 (31.1%)		
N1	62 (26.4%)	35 (29.4%)		
N2	56 (23.8%)	19 (16%)		
N3	46 (19.6%)	28 (23.5%)		
Stage pathologic M, n (%)			1.000	0
M0	221 (92.9%)	107 (93%)		
M1	17 (7.1%)	8 (7%)		
Time, median (IQR)	413.5 (209, 745)	477 (279, 795.75)	0.315	14393.5
Risk score, median (IQR)	0.18 (−0.08, 0.52)	0.18 (−0.1, 0.5)	0.982	15354
Age, median (IQR)	67 (58, 73)	68 (57, 74)	0.929	15215.5

### The establishment and verification of the ICD-related lncRNA signature

**FIGURE 3:**
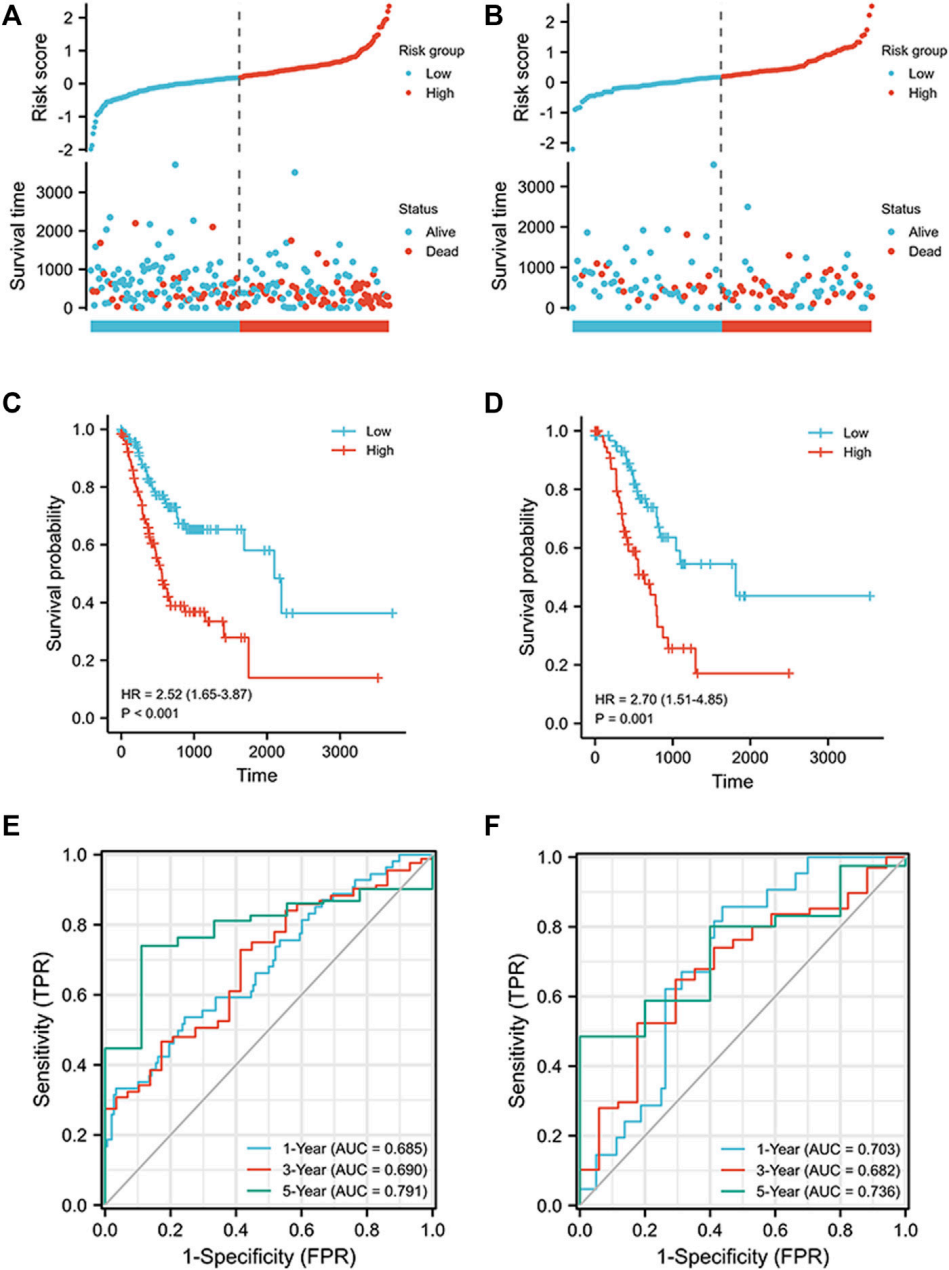
Prognostic analysis of ICD-related lncRNA signature in training and validation cohorts. **(A)** Distribution of risk scores in the training cohort and overall survival status, overall survival, and risk score. **(B)** Distribution of risk scores in the validation cohort and overall survival status, overall survival, and risk score. **(C)** Kaplan–Meier curves for the overall survival of patients in the high- and low-risk groups in the training cohort. **(D)** Kaplan–Meier curves for the overall survival of patients in the high- and low-risk groups in the validation cohort. **(E)** AUC of time-dependent ROC curves verifying the prognostic accuracy of risk scores in the training cohort. **(F)** AUC of time-dependent ROC curves verifying the prognostic accuracy of risk scores in the validation cohort.

The signature was constructed using LASSO regression analysis. The risk score was computed using the following formula: (0.04217 × AL355922.1) + (0.13753 × BASP1-AS1) + (0.04922 × HAGLR) + (−0.08037 × AC005586.1) + (0.18179 × AL391152.1) + (0.48802 × AC129507.1) + (0.61776 × AL354861.3) + (0.0542 × AC037198.1) + (−0.37274 × AP000721.2). We used the median risk score to classify the cohorts into high and low risk groups, as shown in Figure 3A-B. After survival analysis, it was found that the survival probability of the high-risk group was larger than that of the low-risk group, and the difference was statistically significant (training cohort: HR = 2.52 (1.65–3.87), *p* < 0.001; verification cohort: HR = 2.70 (1.51–4.85), *p* < 0.001) (Figure 3C-D). In the training cohort, the AUC reached 0.685 at 1 year, 0.690 at 3 years, and 0.791 at 5 years; in the verification cohort, the AUC reached 0.703 at 1 year, 0.682 at 3 years, and 0.736 at 5 years (Figure 3E-F). These results confirm that the signature has good predictive effectiveness. To further verify the validity of the model, multivariate and univariate regression analyses of the training cohort and the verification cohort were conducted (Tables 2 & 3), and the results show that risk score is an important prognostic factor of gastric cancer.

**TABLE 2:** Univariate and multivariate analyses of risk factors in the training cohort.

Characteristics	Total (N)	Univariate analysis		Multivariate analysis	
		Hazard ratio (95% CI)	*p*-value	Hazard ratio (95% CI)	*p*-value
Risk Score	248	2.812 (2.023–3.908)	**<0.001**	2.688 (1.850–3.904)	**<0.001**
Age	246	1.028 (1.007–1.050)	**0.008**	1.031 (1.009–1.054)	**0.006**
Gender	248				
Male	155	Reference			
Female	93	0.674 (0.431–1.054)	0.084		
Race	214				
White	156	Reference			
Other	8	1.387 (0.553–3.484)	0.486		
Asian	50	0.718 (0.392–1.313)	0.282		
Neoplasm histologic grade	242				
G1-2	91	Reference			
G3	151	1.549 (0.995–2.410)	0.053		
Stage pathologic stage	234				
Stage I–II	105	Reference			
Stage III–IV	129	1.663 (1.074–2.574)	**0.022**	1.571 (1.011–2.441)	**0.045**

**TABLE 3:** Univariate and multivariate analyses of risk factors in the validation cohort.

Characteristic	Total(N)	Univariate analysis		Multivariate analysis	
		Hazard ratio (95% CI)	*p*-value	Hazard ratio (95% CI)	*p*-value
Risk score	124	1.792 (1.157–2.776)	**0.009**	1.759 (1.123–2.756)	**0.014**
Age	123	1.016 (0.988–1.046)	0.260		
Gender	124				
Male	84	Reference			
Female	40	0.983 (0.550–1.758)	0.953		
Race	109				
White	82	Reference			
Asian	23	0.595 (0.233–1.520)	0.278		
Other	4	1.921 (0.589–6.265)	0.279		
Neoplasm histologic grade	121				
G1-2	53	Reference			
G3	68	1.055 (0.598–1.861)	0.854		
Stage event pathologic stage	115				
Stage I–II	56	Reference			
Stage III–IV	59	2.416 (1.288–4.533)	**0.006**	2.303 (1.225–4.331)	**0.010**

### Construction of a new nomogram with clinicopathological information

**FIGURE 4:**
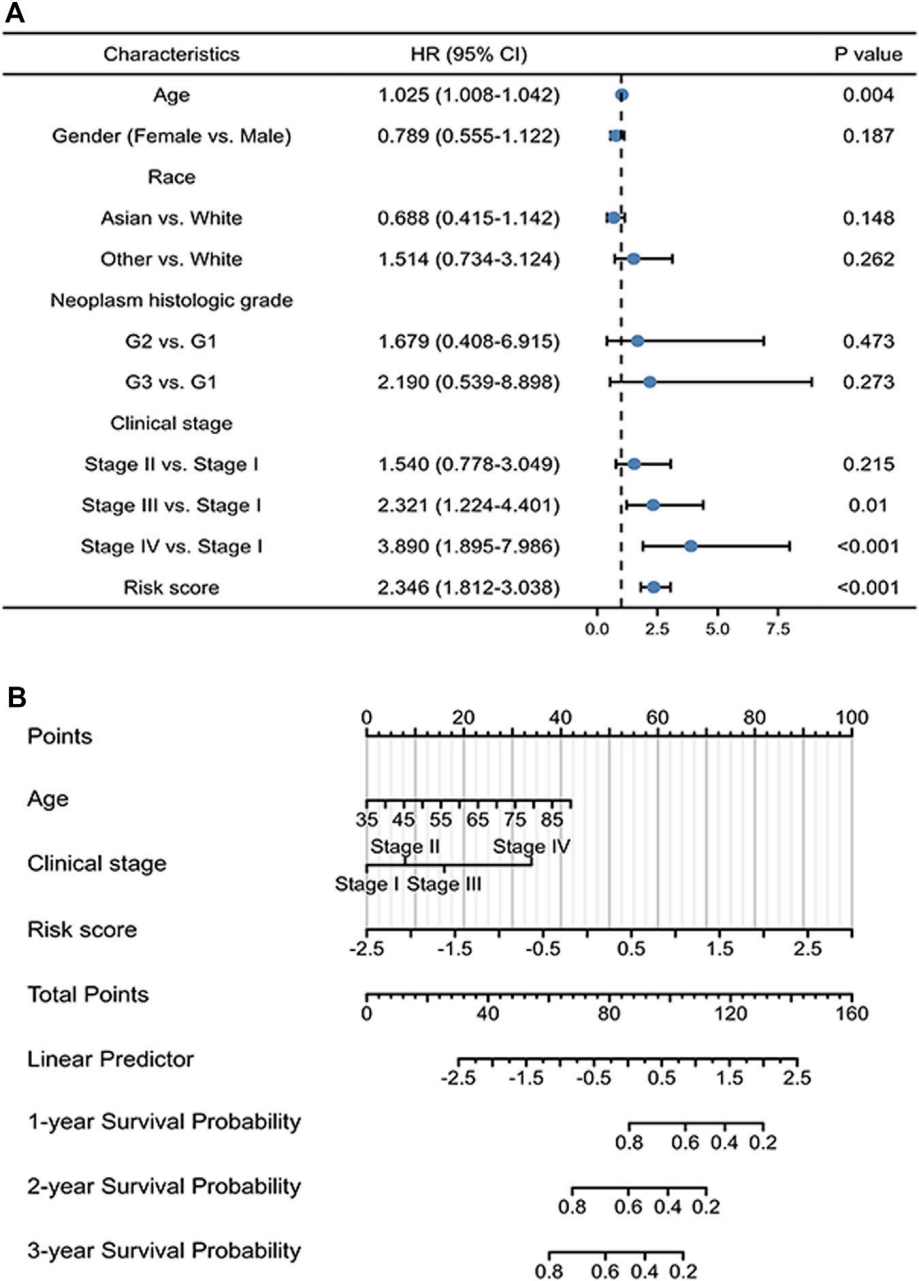
Prognostic value of the ICD-related lncRNA signature. **(A)** Multivariate Cox regression of patient characteristics and the signature as a whole. **(B)** Nomogram of the risk model with clinical information.

Univariate regression analysis of patients with gastric cancer demonstrates that old age, advanced tumor stage (stages III and IV), and high risk scores have a significant adverse effect on prognosis (Figure 4A). More detailed analysis is required to determine the efficacy of the signature. Accordingly, a nomogram was constructed to further verify the prognostic effect of the signature, based on the regression analysis results. The nomogram includes age, tumor stage, and risk score and can predict the 1-, 3-, and 5-year survival probability of gastric cancer patients with a C-index of 0.703 (0.679–0.727) (Figure 4B).

### Differences in immune microenvironment between high- and low-risk groups

**FIGURE 5:**
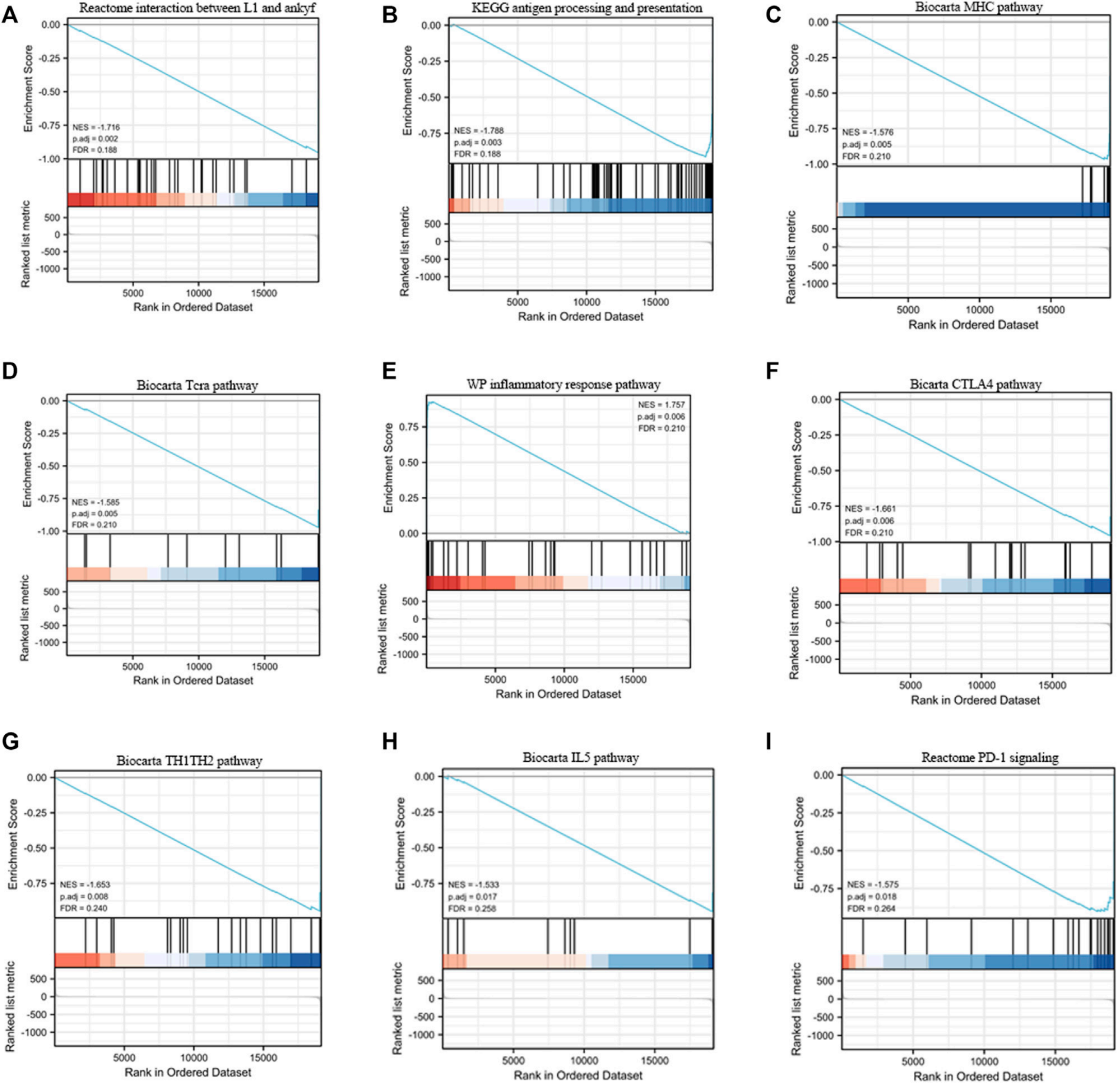
Gene set enrichment analysis (GSEA) of the ICD-related lncRNA prognostic signature. **(A)** Reactome interaction between L1 and ankyrins. **(B)** KEGG antigens and presentation. **(C)** Biocarta MHC pathway. **(D)** Biocarta TCRA pathway. **(E)** WB inflammatory response pathway. **(F)** Biocarta CTLA4 pathway. **(G)** Biocarta Th1Th2 pathway. **(H)** Biocarta IL5 pathway. **(I)** Reactome PD-1 signaling.

The difference in the molecular mechanisms between high- and low-risk groups can be determined by GSEA. In Figure 5, nine immune-related signaling pathways are associated with the signature, including the reactome interaction between L1 and ankyrins, Kyoto Encyclopedia of Genes and Genomes (KEGG) antigen processing and presentation, Biocarta MHC pathway, Biocarta TCRA pathway, WB inflammatory response pathway, Biocarta CTLA4 pathway, Biocarta Th1Th2 pathway, Biocarta IL5 pathway, and reactome PD-1 signaling. These results may provide a theoretical basis for future immunotherapy of gastric cancer.

**FIGURE 6:**
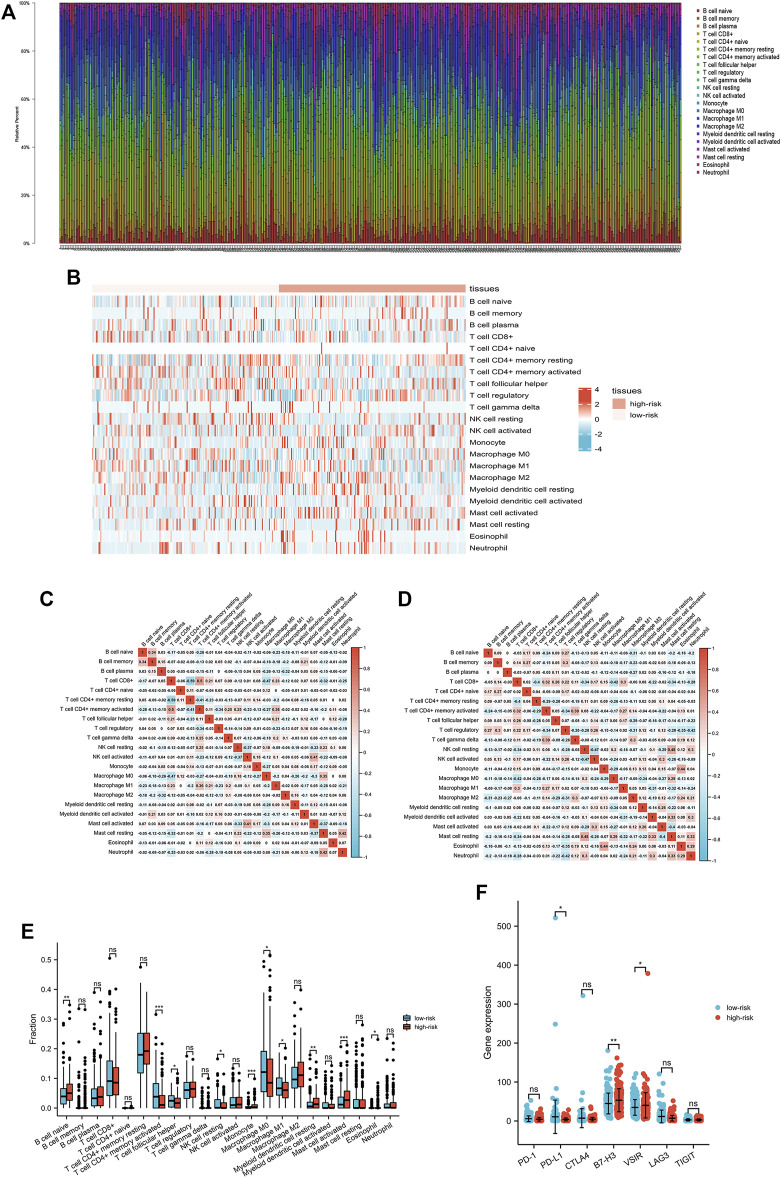
Interactions between the ICD-related lncRNA signature and immune regulation in patients with gastric cancer. **(A)** Degree of immune cell infiltration **(B)** Heatmap of tumor-infiltrating cells in low- and high-risk patients. **(C, D)** Correlation matrix of immune cells in gastric cancer. **(E)** Comparisons of immune cells between low- and high-risk groups. **(F)** Comparison of multiple immune checkpoints between low-risk and high-risk groups, including PD-1, PD-L1, CTLA4, B7-H3, VSIR, LAG3, and TIGIT.

We also studied the TME of patients with gastric cancer because it is closely connected with ICD (1, 13–17). The values obtained from all the procedures were combined, the CIBERSORT algorithms were applied, and the percentage of specific immune cells was estimated, as shown in Figure 6A. The heat map of immune cell expression in the high- and low-risk groups is shown in Figure 6B. The results of correlation analysis of various immune cells are displayed in Figure 6C-E, depicting the differential expression of immune cells that have invaded tumors in patients with gastric cancer. The expression level of naive B cells, monocytes, resting myeloid dendritic cells, activated mast cells, and eosinophils was higher in patients than that in the low-risk group. The expression level of M0 and M1 macrophages, resting NK cells, follicular helper T cells, and activated CD4^**+**^ memory T cells was higher in patients than in the high-risk group. In addition, we examined the expression patterns of diverse immunological checkpoints in the high-risk and low-risk groups, and found that the difference between PD-L1 and VSIR was statistically significant (Figure 6F).

## Discussion

ICD is a special type of cell death that has been found to connect tumor cells with the host’s immune system. ICD can activate the immune system by releasing DAMPs, exerting potent anti-tumor effects, and potentially inducing long-lasting anti-tumor immunity in patients (Feng et al., 2022). ICD is a cell death mode with promising therapeutic prospects for gastric cancer (Liao et al., 2022), and identifying an ICD-related signature could pave the way for more effective treatments of gastric cancer. In our study, we obtained complete RNA-seq and clinical data of 372 patients from the TCGA. By reviewing the existing literature and applying univariate analysis, we identified 138 ICD-related genes, and of these, 11 were prognosis-related. Next, 43 prognosis-related lncRNAs were obtained through Pearson correlation analysis and univariate COX regression analysis, and nine prognosis-related ICD-related lncRNAs were obtained through differential expression analysis and LASSO regression analysis to construct the signature. Further validation was performed through differential expression analysis in gastric cancer tissues and adjacent normal tissues. We found that the high-risk groups stratified by the signature had a better prognosis than the low-risk groups, and that the signature had good predictive value for prognosis. Lastly, we plotted K–M curves to predict the 1-, 3-, and 5-year survival of patients based on their risk score combined with their clinical characteristics. This signature helps doctors make better predictions of prognosis, which should help patients with gastric cancer get appropriate treatment.

A growing number of studies in recent years have shown that lncRNAs play an important role in the genesis and progression of many malignancies, as well as in the TME. In our signature, we identified nine lncRNAs, namely, AL355922.1, BASP1-AS1, HAGLR, AC005586.1, AP000721.2, AL391152.1, AC129507.1, AL354861.3, and AC037198.1. We found that BASP1-AS1 was critical for the development and prognosis of melanoma (Li et al., 2021). Additionally, BASP1-AS1 has been shown to significantly affect the proliferation of glioma cells and may be a new target for glioma treatment (Xu et al., 2021). Many studies have indicated that HAGLR exerts an unfavorable effect on the prognosis and treatment of gastrointestinal tumors. It enhances the resistance of gastric cancer to 5-fluorouracil by targeting the glycolysis pathway, which necessitates the development of new drugs (Hu et al., 2022). Furthermore, HAGLR promotes the occurrence and development of liver cell cancer (HCC) through miR-6785-5p, resulting in a poor prognosis (Li et al., 2020). Similarly, high HAGLR expression in colon cancer accelerates its progression (Sun et al., 2020). LncRNA AL391152.1 has been studied in gastric cancer, and results indicated that AL391152.1 was a novel glycolysis-related lncRNA that could accurately predict the overall survival time of gastric cancer patients (Zeng et al., 2022a). It has also been demonstrated that AL391152.1 is related to cellular aging and can effectively predict the response to immunotherapy in gastric cancer patients (Zeng et al., 2022b). In addition, another study used AL391152.1 to construct a gastric cancer risk score model (Liu et al., 2020). Together, these studies demonstrate that lncRNA AL391152.1 has broad prospects for application. Many studies have found that AC129507.1 plays a role in the development, immunotherapy, and prognosis of gastric cancer, which is consistent with our findings in the present research. Platelet activation-related lncRNA AC129507.1 can serve as a biomarker for prognosis and for the response of gastric cancer patients to immunotherapy (Yuan et al., 2022). Similar findings have been reported in other studies (Han et al., 2021; Zeng et al., 2022b). Three hypoxia-related lncRNA AC037198.1-associated molecular subtypes characterized by different prognoses and immune conditions have been identified, which can provide a theoretical basis for the improvement of clinical diagnosis and treatment of gastric cancer (Fan et al., 2022).

ICD-related lncRNAs have only been studied in liver cancer and stomach adenocarcinoma. He et al. screened 20 lncRNAs based on 33 ICD-related genes to construct a prognostic model. The model was helpful in classifying subtypes of liver cancer based on ICD-related lncRNAs and molecules, and for predicting the prognosis of patients with liver cancer and their therapeutic response to immunotherapy (He et al., 2022). Ding et al. screened five lncRNAs based on 34 ICD-related genes to construct a prognostic model for gastric adenocarcinoma. The model could predict the cumulative survival rate and guide individual treatment (Ding et al., 2022). The aforementioned studies all indicated that ICD was closely related to lncRNAs and to disease prognosis. In the present research, we constructed a different prognostic model based on the 138 ICD-related genes identified in the latest studies, and consistent results were obtained.

The immune system of cancer patients constantly fights cancer cells. As a result, some cancer cells have developed the ability to evade recognition and elimination by the immune system, which is referred to as “immune evasion” (Wang et al., 2022). Gastric cancer cells can avoid monitoring and attack by the immune system (Schreiber et al., 2011). Immune evasion is closely associated with the immune microenvironment, which could become a new aspect of research on gastric cancer treatment (Wang et al., 2021b; Ding et al., 2022; Gao et al., 2022; Han et al., 2022; Liao et al., 2022; Xu et al., 2022). Therefore, we analyzed the effect of ICD-related gene mutations on immune cell infiltration in the high- and low-risk groups and found that there were statistically significant differences between the two groups in many immune cells. This finding demonstrates the effectiveness of the constructed risk model for guiding future immunotherapy regimens and the development of immunoreactive drugs. In addition, various signaling pathways, which may play a crucial role in the immune evasion of gastric cancer cells, could significantly affect the prognosis and drug resistance of gastric cancer patients. Therefore, we also performed a GSEA analysis on the high- and low-risk groups, and the results provide a new perspective for future signal pathway-related gastric cancer treatment.

Because the immune system plays a critical role in tumor development, scholars are seeking to extend the survival of patients with gastric cancer through innovative immunotherapy (Zhang et al., 2021). One of the most recent therapeutic strategies combines immunotherapy with immuno-checkpoint inhibitor and ICD-related therapies. Significant progress has been made in immuno-checkpoint inhibitors, and several drugs based on immune checkpoints have been developed in recent years (Zou et al., 2019). We found that certain immunological checkpoints (B7-H3 and VSIR) were overexpressed in high-risk patients. However, many individuals responded poorly to this therapeutic method, and some showed resistance. ICD-related therapy may bring new opportunities for the treatment of refractory gastric cancer.

Our research has some limitations. First, it is a bioinformatics analysis based on public databases. Although we downloaded samples from multiple databases, the data are still limited. Second, this study is a retrospective study, and the findings still need to be verified by further multicenter prospective cohort studies with large sample sizes.

## Conclusion

We constructed a signature of nine ICD-related lncRNAs based on ICD-related genes and demonstrated its effectiveness in predicting the prognosis of patients with gastric cancer. The signature is strongly associated with the immune microenvironment, and our findings support a new vision and direction for immunotherapy in the treatment of gastric cancer.

## Data Availability

The original contributions presented in the study are included in the article/Supplementary Material. Further inquiries can be directed to the corresponding authors.

## Abbreviations

ANOVA, one-way analysis of variance; AUC, area under curve; GSEA, gene set enrichment analysis; LASSO, least absolute shrinkage and selection operator; lncRNAs, long non-coding RNAs
